# Oral curcumin ameliorates acute murine campylobacteriosis

**DOI:** 10.3389/fimmu.2024.1363457

**Published:** 2024-05-24

**Authors:** Markus M. Heimesaat, Soraya Mousavi, Fábia Daniela Lobo de Sá, Elisa Peh, Jörg-Dieter Schulzke, Roland Bücker, Sophie Kittler, Stefan Bereswill

**Affiliations:** ^1^ Gastrointestinal Microbiology Research Group, Institute of Microbiology, Infectious Diseases and Immunology, Charité - Universitätsmedizin Berlin, Corporate Member of Freie Universität Berlin, Humboldt-Universität zu Berlin, and Berlin Institute of Health, Berlin, Germany; ^2^ Clinical Physiology/Nutritional Medicine, Department of Gastroenterology, Infectious Diseases and Rheumatology, Charité – Universitätsmedizin Berlin, Corporate Member of Freie Universität Berlin, Humboldt-Universität zu Berlin, and Berlin Institute of Health, Berlin, Germany; ^3^ Institute for Food Quality and Food Safety, University of Veterinary Medicine Hannover, Hannover, Germany

**Keywords:** curcumin, polyphenols, preclinical placebo-controlled intervention study, *Campylobacter jejuni*, secondary abiotic IL-10 -/-mice, acute enterocolitis, campylobacteriosis model, host-pathogen interaction

## Abstract

**Introduction:**

Human infections with the food-borne enteropathogen *Campylobacter jejuni* are responsible for increasing incidences of acute campylobacteriosis cases worldwide. Since antibiotic treatment is usually not indicated and the severity of the enteritis directly correlates with the risk of developing serious autoimmune disease later-on, novel antibiotics-independent intervention strategies with non-toxic compounds to ameliorate and even prevent campylobacteriosis are utmost wanted. Given its known pleiotropic health-promoting properties, curcumin constitutes such a promising candidate molecule. In our actual preclinical placebo-controlled intervention trial, we tested the anti-microbial and anti-inflammatory effects of oral curcumin pretreatment during acute experimental campylobacteriosis.

**Methods:**

Therefore, secondary abiotic IL-10^-/-^ mice were challenged with synthetic curcumin via the drinking water starting a week prior oral *C. jejuni* infection. To assess anti-pathogenic, clinical, immune-modulatory, and functional effects of curcumin prophylaxis, gastrointestinal *C. jejuni* bacteria were cultured, clinical signs and colonic histopathological changes quantitated, pro-inflammatory immune cell responses determined by *in situ* immunohistochemistry and intestinal, extra-intestinal and systemic pro-inflammatory mediator measurements, and finally, intestinal epithelial barrier function tested by electrophysiological resistance analysis of colonic *ex vivo* biopsies in the Ussing chamber.

**Results and discussion:**

Whereas placebo counterparts were suffering from severe enterocolitis characterized by wasting symptoms and bloody diarrhea on day 6 post-infection, curcumin pretreated mice, however, were clinically far less compromised and displayed less severe microscopic inflammatory sequelae such as histopathological changes and epithelial cell apoptosis in the colon. In addition, curcumin pretreatment could mitigate pro-inflammatory innate and adaptive immune responses in the intestinal tract and importantly, rescue colonic epithelial barrier integrity upon *C. jejuni* infection. Remarkably, the disease-mitigating effects of exogenous curcumin was also observed in organs beyond the infected intestines and strikingly, even systemically given basal hepatic, renal, and serum concentrations of pro-inflammatory mediators measured in curcumin pretreated mice on day 6 post-infection. In conclusion, the anti-*Campylobacter* and disease-mitigating including anti-inflammatory effects upon oral curcumin application observed here highlight the polyphenolic compound as a promising antibiotics-independent option for the prevention from severe acute campylobacteriosis and its potential post-infectious complications.

## 1 Introduction

Human infections with the food-borne enteropathogen *Campylobacter jejuni* are responsible for increasing incidences of acute campylobacteriosis cases worldwide and are of both, tremendous medical and financial impact (1). In 2021, over 127,000 new campylobacteriosis infections were reported in Europe but the real case numbers including undiagnosed and/or non-reported illnesses are estimated to exceed the reported ones several-fold (2). Within the *Campylobacteraceae* family, the Gram-negative, microaerophilic, non-spore-forming *C. jejuni* bacteria live commensally in the intestinal tracts of warm-blooded vertebrate species, including birds, usually generating no symptoms (3, 4). However, food chain transmission via contaminated undercooked meat from poultry or other livestock, unpasteurized milk, and its byproducts, as well as surface water may all be considered as potential infectious sources upon ingestion by humans (5). After a successful gastro-duodenal passage, the highly motile *C. jejuni* bacteria invade the distal intestinal tissues, and specific bacterial cell wall components, such as the endotoxin lipo-oligosaccharide (LOS), induce the Toll-like receptor-4 (TLR-4)-dependent hyperactivation of the host immune system (6–9). In this pro-inflammatory immune cascade, both innate and adaptive immune cell subsets, including neutrophils and T cells, respectively, are recruited to the infection site, and pro-inflammatory mediators including interferon-gamma (IFN-γ), tumor necrosis factor-alpha (TNF-α), interleukin (IL)-6, and nitric oxide (NO) are released to restrict the infection, but by the expenses of exerting oxidative stress and damage to the intestinal tissues (10). This results in epithelial cell damage, induction of apoptosis, ulcerations, crypt drop-outs, and crypt abscesses, which can lead to malabsorptive dysfunctions and to the barrier-impaired “leaky gut” (11, 12). Depending on the individual immunological fitness of the human host as well as the distinct virulence genes expressed by the pathogen, infected humans may present with symptoms of different severities after an incubation period ranging from 2 to 6 days. For instance, patients may complain about general malaise, nausea, vomiting, abdominal cramps, watery or even bloody diarrhea, mucous discharge, and fever (13, 14). Typically, patients recover completely two weeks post-infection (p.i.). However, rarely, post-infectious autoimmune morbidities affecting the intestinal tract (i.e., irritable bowel syndrome (IBS), chronic inflammatory bowel disease (IBD)), the joints (i.e., reactive arthritis (RA)), and the central nervous system (i.e., Guillain Barré syndrome (GBS)) may appear weeks to months after the initial infection (14–16). It is interesting to note that the chance of developing post-infectious sequelae is strongly correlated with the severity of the previous enteritis episode, which in turn, depends on the sialylation state of the *C. jejuni*-LOS (17). Patients with campylobacteriosis are typically treated with symptomatic therapies such as analgesic, antipyretic, and spasmolytic medications, along with electrolyte replacement and rehydration. Patients with severe medical conditions, such as those with immune-suppressive disorders, may be treated with antibiotics including ciprofloxacin or erythromycin (14, 18). The increasing prevalence of infections with multi-drug resistant *C. jejuni* strains, however, can make it rather challenging to effectively treat severe campylobacteriosis cases in critically ill patients (18). Thus, it is imperative to find non-toxic, antibiotic-independent therapeutic approaches to ameliorate and even prevent acute campylobacteriosis and its post-infectious sequelae.

Our One Health approach to identifying new protective substances against campylobacteriosis focused on curcumin. The bright yellowish curcumin can be found in the roots of the turmeric plant *Curcuma longa* belonging to the ginger family and is responsible for the spicy taste of curry spices. For a long time, the polyphenolic compound has been known for its health-promoting properties and is highly appreciated in traditional medicine as remedy for many different morbidities (19). Both *in vitro* and *in vivo* studies provided evidence for potent anti-inflammatory, anti-oxidant, anti-infectious, and even anti-tumor effects of exogenous curcumin as reviewed previously (20, 21). A recent *in vitro* study revealed that curcumin was able to compromise the quorum sensing of *C. jejuni*, which is essentially involved in bacterial motility, colonization, and interaction with epithelial cells (22). Notably, oral curcumin intake could alleviate acute experimental inflammation in the small and large intestinal tract (23–25). In several randomized clinical trials, curcumin has been even shown efficient in maintaining remission in patients suffering from ulcerative colitis when administered in combination with mesalazine (26–28).

Given its known pleiotropic disease-ameliorating properties, curcumin constitutes a promising candidate molecule for combatting campylobacteriosis. This prompted us to test curcumin for its potential anti-microbial and anti-inflammatory effects during *C. jejuni*-induced enterocolitis in mice. Two prerequisites need to be taken into consideration, however. To enable *C. jejuni* to colonize stably the murine intestinal tract, mice need to be pretreated with antibiotics to deplete the commensal gut microbiota providing a protective colonization resistance against *C. jejuni* (29, 30). Furthermore, wildtype mice are known to be approximately 10,000 times more resistant to TLR-4 ligands such as lipo-polysaccharides (LPS) and LOS as compared to humans (31), whereas the *il10* gene knock-out can make mice vulnerable to *C. jejuni*-LOS, however (8). Secondary abiotic (SAB) IL-10^-/-^ mice, in which the intestinal microbiota had been depleted by preceding antibiotic treatment, were shown to develop acute enterocolitis with bloody diarrhea and wasting symptoms within less than a week after oral *C. jejuni* infection. Moreover, the animals generate pro-inflammatory immune responses that did not only impact the intestinal tract but also extra-intestinal organs such as the liver, kidneys, and systemic circulation (32–34). Our previous studies underscored that the SAB IL-10^-/-^ mouse model is a reliable way to assess the potential anti-microbial and disease-mitigating effects of defined compounds including phenolic molecules such as carvacrol (35, 36) and resveratrol (37, 38), for instance, during acute campylobacteriosis. In this preclinical placebo-controlled investigation, we administered synthetic curcumin orally as a preventative measure to SAB IL-10^-/-^ mice beginning a week before *C. jejuni* infection. We evaluated the effects of this regimen on various parameters, including i.) fecal *C. jejuni* shedding over time p.i., ii.) gastrointestinal pathogen loads, iii.) clinical conditions, iv.) microscopic inflammatory alterations in the colonic tissues, and v.) intestinal effects as well as vi.) extra-intestinal outcomes including vii.) systemic pro-inflammatory immune responses on day 6 p.i.

## 2 Materials and methods

### 2.1 Ethics declaration

All mouse studies were approved by the commission for animal experiments led by the “Landesamt für Gesundheit und Soziales” (LaGeSo, Berlin, registration numbers G0104/19) and carried out in compliance with the European Guidelines for animal welfare (2010/63/EU). The clinical status of the experimental animals was evaluated daily.

### 2.2 Mice, gut microbiota depletion, curcumin treatment

Under specific pathogen-free (SPF) conditions, both male and female IL-10^-/-^ mice on a C57BL/6j background were raised and kept within the same unit located at the Forschungseinrichtungen für Experimentelle Medizin (FEM, Charité – Universitätsmedizin Berlin). Commensal gut microbiota depletion in mice was achieved by an 8-week treatment with ampicillin plus sulbactam (2 g/L and 1 g/L, respectively; Dr. Friedrich Eberth Arzneimittel, Ursensollen, Germany) via the autoclaved drinking water (*ad libitum*) starting immediately post-weaning, as previously described (34). The day before initiation of the protective treatment, the antibiotics were stopped to ensure drug washout (i.e., day -8).

Seven days prior the *C. jejuni* infection (i.e., day -7), treatment with curcumin (from Sigma-Aldrich, München, Germany) was initiated. To improve water solubility, the synthetic compound was dissolved in 2% carboxy-methylcellulose (Sigma-Aldrich, München, Germany) which resulted in a final concentration of 0.05%. The curcumin suspensions were finally concentrated to 1.0 mg/mL resulting in daily treatment dosages of 200 mg per kilogram of body weight, equivalent to the concentration applied in acute murine ileitis previously (23). Placebo treated (and subsequently infected) mice received vehicle only (positive controls). Furthermore, naive (i.e., untreated and non-infected mice) served as negative controls.

### 2.3 *C. jejuni* infection and gastrointestinal colonization properties

On days 0 and 1, 10^9^ colony-forming units (CFU) of the *C. jejuni* 81–176 strain were applied to sex- and age-matched litter-mate mice that were three months old by oral gavage. Animals were handled under rigorous aseptic circumstances and kept in a sterile environment (autoclaved food and drinking solutions). *C. jejuni* were quantitatively evaluated in feces over time p.i. and in luminal samples obtained from different regions of the gastrointestinal tract (specifically from the stomach, duodenum, ileum, and colon) at day 6 p.i. by culture, as previously reported (29, 39) allowing for the assessment of gastrointestinal colonization features. The viable pathogen detection limit was 100 CFU/g.

### 2.4 Clinical outcome

The clinical conditions of the mice were evaluated before and after the infection with *C. jejuni* using a standardized campylobacteriosis score (12 points maximum). This score addressed the stool consistency (0: formed feces; 2: pasty feces; 4: liquid feces), the abundance of blood in stool (0: no blood; 2: microscopic detection of blood by the Guajac method using Haemoccult, Beckman Coulter/PCD, Krefeld, Germany; 4: macroscopic blood visible), and the overall clinical aspect (0: normal; 2: ruffled fur, less locomotion; 4: isolation, severely compromised locomotion, pre-final aspect), as previously described (40).

### 2.5 Sampling

Mice were necrotized by CO_2_ inhalation on day 6 p.i. Under sterile conditions, luminal gastrointestinal samples (i.e., from the stomach, duodenum, ileum, and colon) and *ex vivo* biopsies were obtained from the colon, mesenteric lymph nodes (MLN), liver, and kidneys. Cardiac blood was drawn for cytokine testing in serum samples. Each mouse had its colon sample taken in parallel for studies involving microbiology, immunohistopathology, and immunology. A ruler was used to measure the large intestinal lengths (in cm).

### 2.6 Immunohistochemistry

Colonic *ex vivo* samples that had been instantly fixed in 5% formalin and embedded in paraffin, were used for *in situ* immunohistochemical investigations. In summary, paraffin slices of *ex vivo* biopsies originating from the colon (5 µm) were stained with primary antibodies directed against cleaved caspase 3 (Asp175, Cell Signaling, Beverly, MA, USA, 1:200), MPO7 (No. A0398, Dako, Glostrup, Denmark; 1:500), CD3 (#N1580, Dako, Glostrup, Denmark; 1:10), and B220 (No. 14–0452-81, eBioscience, San Diego, CA, USA; 1:200), respectively as reported earlier (41). Following that, positively labeled cells were analyzed in a blinded fashion using light microscopy at a 400-times magnification and the median number from 6 high-power fields (HPF, 0.287 mm^2^) per mouse specimen were calculated.

### 2.7 Pro-inflammatory mediators

Colonic explants were cut lengthwise, cleaned in phosphate-buffered saline (PBS; Thermo Fisher Scientific, Waltham, MA, USA), and ~1 cm^2^ of the tissue strips in addition to *ex vivo* biopsies derived from the liver (~1 cm^3^) and kidney (half of the organ after longitudinal cut) then placed in 24-flat-bottom well culture plates (Thermo Fisher Scientific, Waltham, MA, USA) containing 500 µL of serum-free RPMI 1640 medium (Thermo Fisher Scientific, Waltham, MA, USA) plus 100 µg/mL of streptomycin and 100 IU/mL of penicillin (both from Biochrom, Berlin, Germany). Following eighteen hours at 37°C, the corresponding culture supernatants and serum samples were subjected to the Mouse Inflammation Cytometric Bead Assay (CBA; BD Biosciences, Heidelberg, Germany) using a BD FACSCanto II flow cytometer to measure IFN-γ, TNF-α, monocyte chemoattractant protein-1 (MCP-1), and IL-6. NO was measured with the Griess reaction (42).

### 2.8 Electrophysiological measurements

*Ex vivo* biopsies from the terminal large intestine were moved to Ussing chambers (remaining unstripped; 0.049 cm^2^ area). An automatic clamp device (CVC6, Fiebig Hard & Software, Berlin, Germany) was applied to measure the transmural electrical resistance (*R*_t_) for one hour at 37°C under voltage clamp conditions. The bathing solution that was equilibrated with carbogen gas (pH 7.4) had the following composition: Beta-hydroxybutyric acid (0.5 mmol/L), L-glutamine (2.5 mmol/L), D(+)-mannose (10.0 mmol/L), D(+)-glucose (10.0 mmol/L), NaCl (113.6 mmol/L), KCl (5.4 mmol/L), CaCl_2_ (1.2 mmol/L), MgCl_2_ (1.2 mmol/L), Na_2_HPO_4_ (2.4 mmol/L), NaH_2_PO_4_ (0.6 mmol/L), and NaHCO_3_ (21.0 mmol/L).

### 2.9 Statistical analysis

GraphPad Prism (version 9; San Diego, CA, USA) was used to determine medians and significance levels following the pooling of data from three separate trials. The Anderson-Darling test was applied to evaluate the normalization of data sets. Pairwise comparisons of regularly distributed and non-normally distributed data were performed using the Student’s t-test and the Mann-Whitney test, respectively. The one-way ANOVA with Tukey post-hoc test (for regularly distributed data) and the Kruskal-Wallis test with Dunn’s post-hoc test (for non-normally distributed data) were used for multiple comparisons. Significant two-sided probability (p) values were defined as < 0.05.

## 3 Results

### 3.1 Gastrointestinal pathogen loads following curcumin pretreatment of *C. jejuni* infected mice

**Figure 1:**
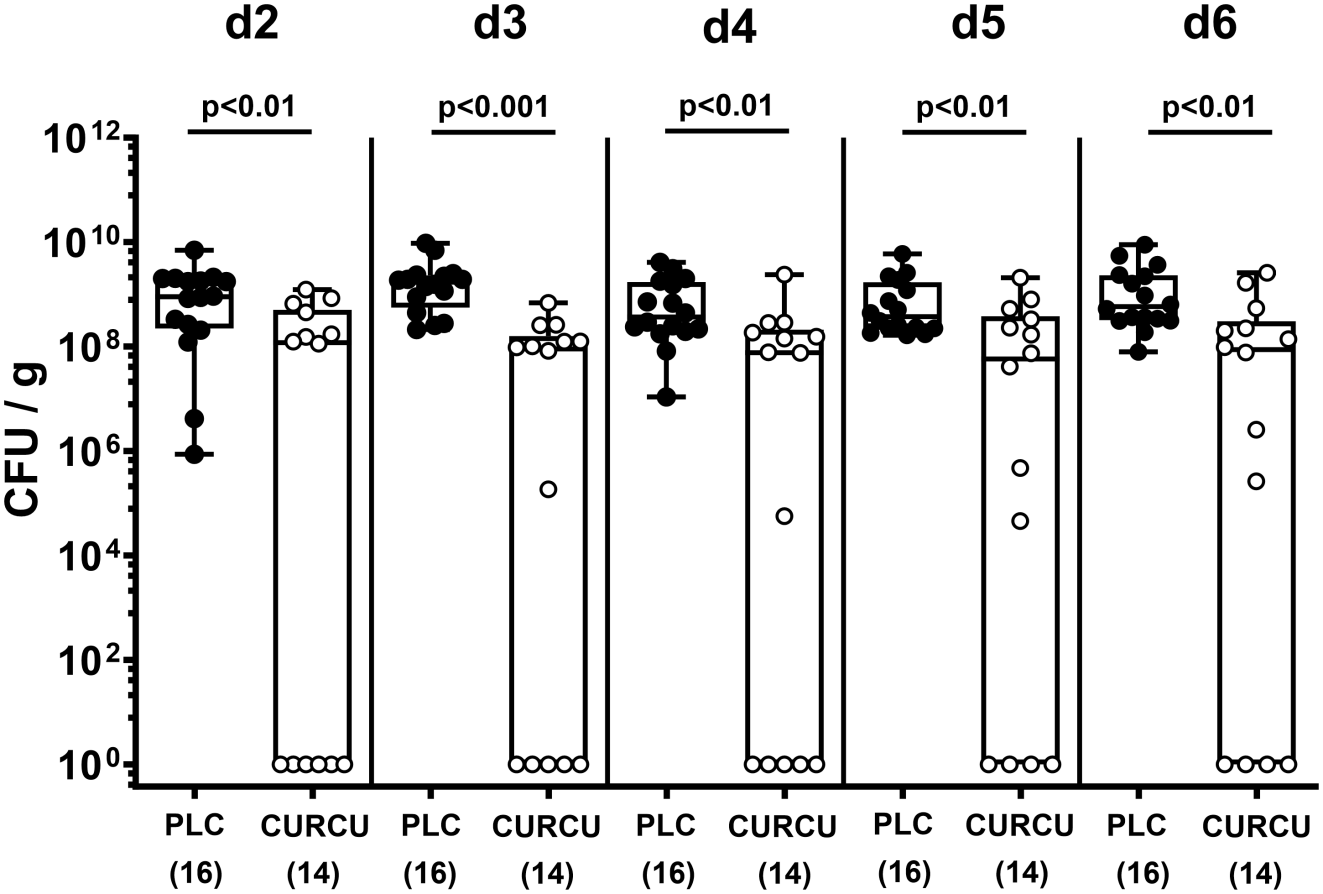
Fecal pathogen shedding over time following *C. jejuni* infection of mice with curcumin prophylaxis. Secondary abiotic IL-10^-/-^ mice were pretreated with curcumin (CURCU; open circles) or placebo (PLC; closed circles) via the drinking water starting 7 days prior peroral infection with *C. jejuni* 81–176 strain on day (d) 0 and d1. Fecal *C. jejuni* numbers were determined daily post-infection by culture (in colony forming units per gram; CFU/g). Box plots (25^th^ and 75^th^ percentiles), whiskers (minimum and maximum values), medians (black bar in boxes), numbers of analyzed mice pooled from 3 independent experiments (in parentheses), and significance levels (p values) determined by the Mann-Whitney test are indicated.

**Figure 2:**
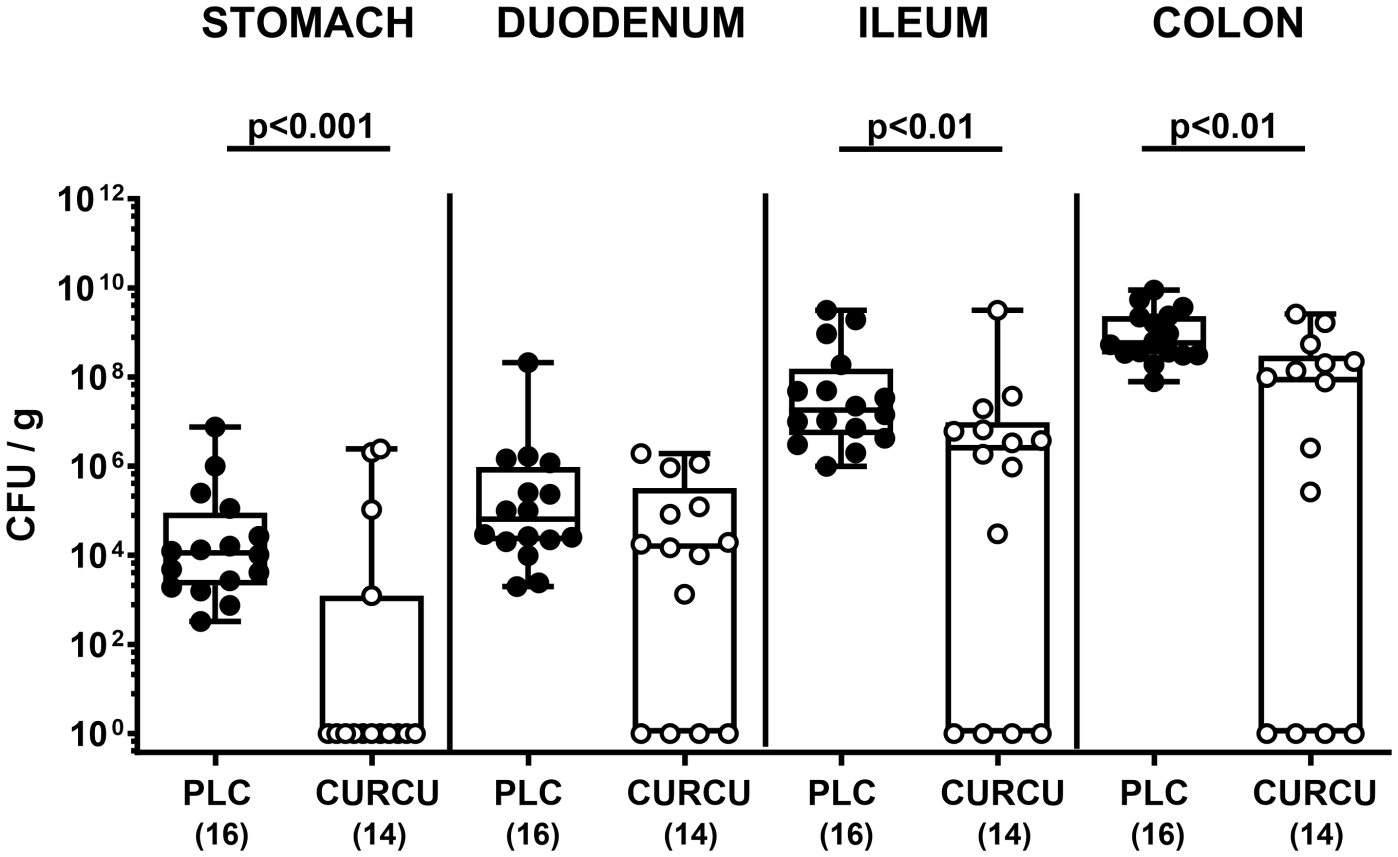
Gastrointestinal pathogen counts following *C. jejuni* infection of mice with curcumin prophylaxis. Secondary abiotic IL-10^-/-^ mice were pretreated with curcumin (CURCU; open circles) or placebo (PLC; closed circles) via the drinking water starting 7 days prior peroral infection with *C. jejuni* 81–176 strain on days 0 and 1. At necropsy (i.e., day 6 post-infection), luminal *C. jejuni* bacteria were quantified in the gastrointestinal tract (as indicated) by culture (in colony forming units per gram; CFU/g). Box plots (25^th^ and 75^th^ percentiles), whiskers (minimum and maximum values), medians (black bar in boxes), numbers of analyzed mice pooled from 3 independent experiments (in parentheses), and significance levels (p values) determined by the Mann-Whitney test are indicated.

Seven days following initiation of the oral curcumin application, SAB IL-10^-/-^ mice were infected with 10^9^ viable *C. jejuni* bacteria on days 0 and 1 by gavage. To test the effects of exogenous curcumin on the enteropathogenic colonization of the intestinal tract, we determined the fecal *C. jejuni* loads by culture every day following infection. As early as 24 hours after the second oral *C. jejuni* challenge (i.e., on day 2 p.i.), the pathogens could be isolated from the feces of placebo controls at high median loads of 10^9^ CFU per g, whereas curcumin pretreated mice exhibited approximately one order of magnitude lower median fecal *C. jejuni* bacteria numbers as compared to the latter which also held true for days 3 until 6 p.i. (p<0.01–0.001; **Figure 1**). At necropsy we further quantified luminal *C. jejuni* bacteria in distinct gastrointestinal regions. Whereas in curcumin pretreated mice approximately one order of magnitude lower median pathogen numbers could be determined in the ileal and colonic lumen if compared to placebo counterparts (p<0.01), even 4 log orders fewer median bacterial cells were isolated from the stomach of the former as compared to the latter on day 6 p.i. (p<0.001; **Figure 2**). Hence, curcumin pretreatment of *C. jejuni* infected mice resulted in lower gastrointestinal pathogen loads.

### 3.2 Inflammatory sequelae upon curcumin pretreatment of *C. jejuni* infected mice

**Figure 3:**
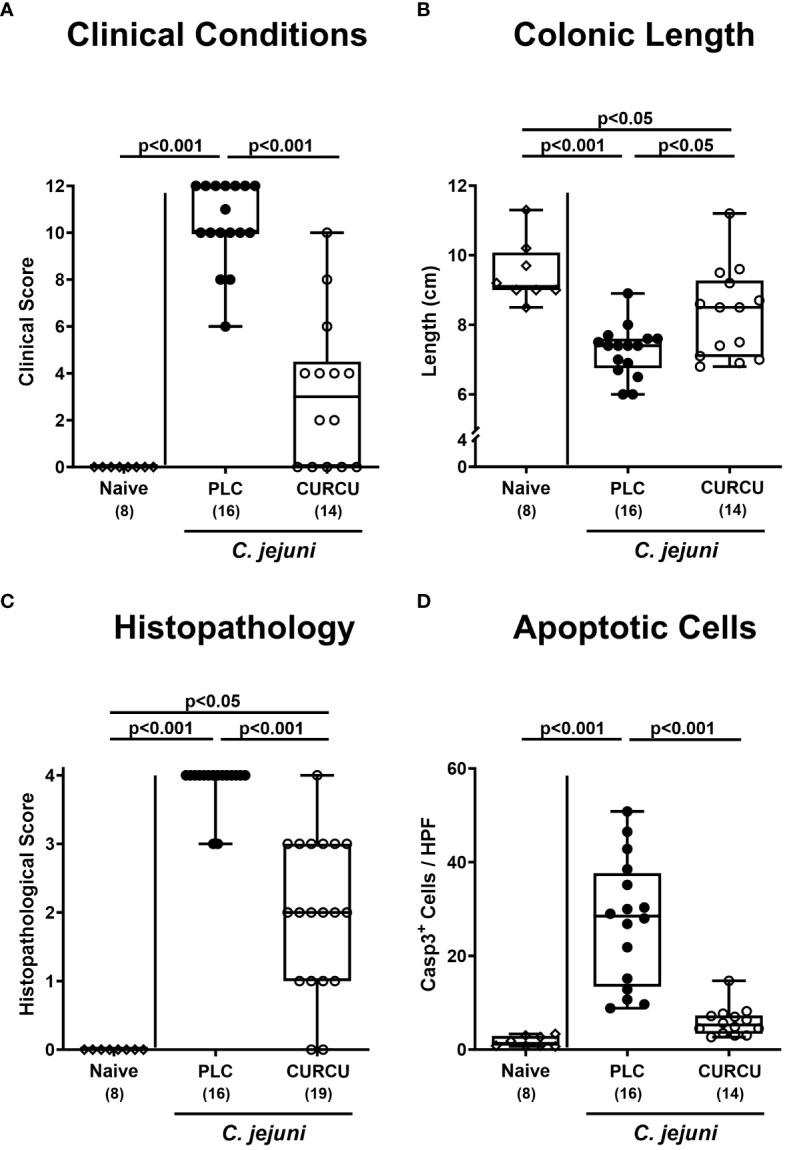
Macroscopic and microscopic inflammatory signs following curcumin pretreatment of *C*. *jejuni* infected mice. Secondary abiotic IL-10^-/-^ mice were pretreated with curcumin (CURCU; open circles) or placebo (PLC; closed circles) via the drinking water starting 7 days prior peroral infection with *C*. *jejuni* 81–176 strain on days 0 and 1. The macroscopic inflammatory signs including **(A)** the clinical conditions as quantified with a campylobacteriosis scoring system (see methods) and **(B)** the colonic lengths as measured with a ruler (in cm) were surveyed on day 6 post-infection. Furthermore, the microscopic inflammatory changes were quantitatively assessed in colonic paraffin sections with **(C)** histopathological scores (see methods) and **(D)** average numbers of apoptotic epithelial cells (positive for caspase3, Casp3) from 6 high power fields (HPF, 400-times magnification) per animal. Naive mice (open diamonds) served as non-infected controls without prophylaxis. Box plots (25^th^ and 75^th^ percentiles), whiskers (minimum and maximum values), medians (black bar in boxes), numbers of analyzed mice pooled from 3 independent experiments (in parentheses), and significance levels (p values) determined by the Kruskal-Wallis test with Dunn’s post-hoc test **(A, C)** or by the one sided ANOVA test with Tukey’s *post-hoc* test **(B, D)** are indicated.

Furthermore, we tested the impact of curcumin prophylaxis on the clinical course of *C. jejuni* infected mice with clinical campylobacteriosis scores assessing the overall clinical aspect and severity of bloody diarrhea. Whereas on day 6 p.i. placebo control animals displayed highly elevated clinical scores indicative for severe acute campylobacteriosis (p<0.001 versus naive), clinical scores were lower in curcumin pretreated mice and did not even differ from basal values (not significant (n.s.) versus naive mice; **Figure 3A**). Remarkably, 35.7% of mice from the verum cohort did not exhibit any clinical signs of *C. jejuni* infection at all (i.e., campylobacteriosis score of 0). Since acute inflammation is known to result in enhanced shrinkage of the affected intestine (32, 43), we measured the colonic lengths in the sacrificed mice. In fact, the colonic lengths were lower in infected as compared to naive mice (p<0.05–0.001), whereas higher values could be obtained in curcumin as compared to placebo pretreated mice on day 6 p.i. (p<0.05; **Figure 3B**), further indicative for mitigated gross *C. jejuni*-induced disease upon oral curcumin prophylaxis.

Moreover, we analyzed potential anti-inflammatory effects of curcumin pretreatment during campylobacteriosis on the microscopic level. Therefore, we quantified the extent of histological cell damage in the infected colon and obtained increased histopathological scores in mice from the placebo and curcumin cohorts (p<0.001 and p<0.05 versus naive, respectively) on day 6 p.i., but with lower values in the latter versus the former (p<0.001; **Figure 3C**). Whereas the histopathological scores in the placebo control group reached mostly maximum values, those obtained from mice of the curcumin cohort varied considerably with reaching the maximum score in a single animal, whereas in two mice no histopathological changes could be observed at all on day 6 p.i. (**Figure 3C**). To further grade the large intestinal inflammation upon enteropathogenic infection, we quantified the apoptotic epithelial cell responses in the colon applying *in situ* immunohistochemistry. Remarkably, *C. jejuni* infection resulted in increased numbers of apoptotic colonic epithelial cells in placebo (p<0.001 versus naive), but not in curcumin pretreated mice on day 6 p.i. (n.s. versus naive; p<0.001 versus placebo; **Figure 3D**). Hence, oral curcumin prophylaxis also mitigated *C. jejuni*-induced histopathological and apoptotic cell damage in the colon.

### 3.3 Colonic immune cell responses following curcumin pretreatment of *C. jejuni* infected mice

**Figure 4:**
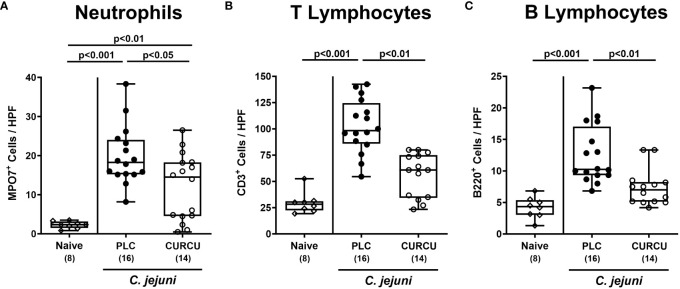
Colonic immune cell responses following curcumin pretreatment of *C*. *jejuni* infected mice. Secondary abiotic IL-10^-/-^ mice were pretreated with curcumin (CURCU; open circles) or placebo (PLC; closed circles) via the drinking water starting 7 days prior peroral infection with *C*. *jejuni* 81–176 strain on days 0 and 1. On day 6 post-infection, the average numbers of **(A)** neutrophils (MPO7^+^), **(B)** T lymphocytes (CD3^+^), and **(C)** B lymphocytes (B220^+^) were determined in the colonic mucosa and lamina propria from 6 high power fields (HPF, 400-times magnification) per animal in immunohistochemically stained paraffin sections. Naive mice (open diamonds) served as non-infected controls without prophylaxis. Box plots (25^th^ and 75^th^ percentiles), whiskers (minimum and maximum values), medians (black bar in boxes), numbers of analyzed mice pooled from 3 independent experiments (in parentheses), and significance levels (p values) determined by the Kruskal-Wallis test with Dunn’s *post-hoc* test are indicated.

To analyze the effects of curcumin pretreatment on *C. jejuni*-induced immune cell responses, we stained colonic paraffin sections with antibodies against surface markers of defined innate and adaptive immune cell subsets. On day 6 p.i., elevated numbers of MPO7^+^ neutrophilic granulocytes were detected in the colonic mucosa and lamina propria (p<0.01–0.001 versus naive), but with lower counts in curcumin as compared to placebo pretreated mice (p<0.05; **Figure 4A**). When analyzing colonic CD3^+^ T and B220^+^ B lymphocyte populations, *C. jejuni*-induced increases could exclusively be observed in placebo controls (p<0.001 versus naive), whereas curcumin prophylaxis resulted in basal adaptive immune cell counts in the colon on day 6 p.i. (n.s. versus naive; **Figures 4B, C**). Hence, curcumin prophylaxis mitigated *C. jejuni-*induced innate and adaptive immune responses in the colon.

### 3.4 Intestinal pro-inflammatory mediator secretion following curcumin pretreatment of *C. jejuni* infected mice

**Figure 5:**
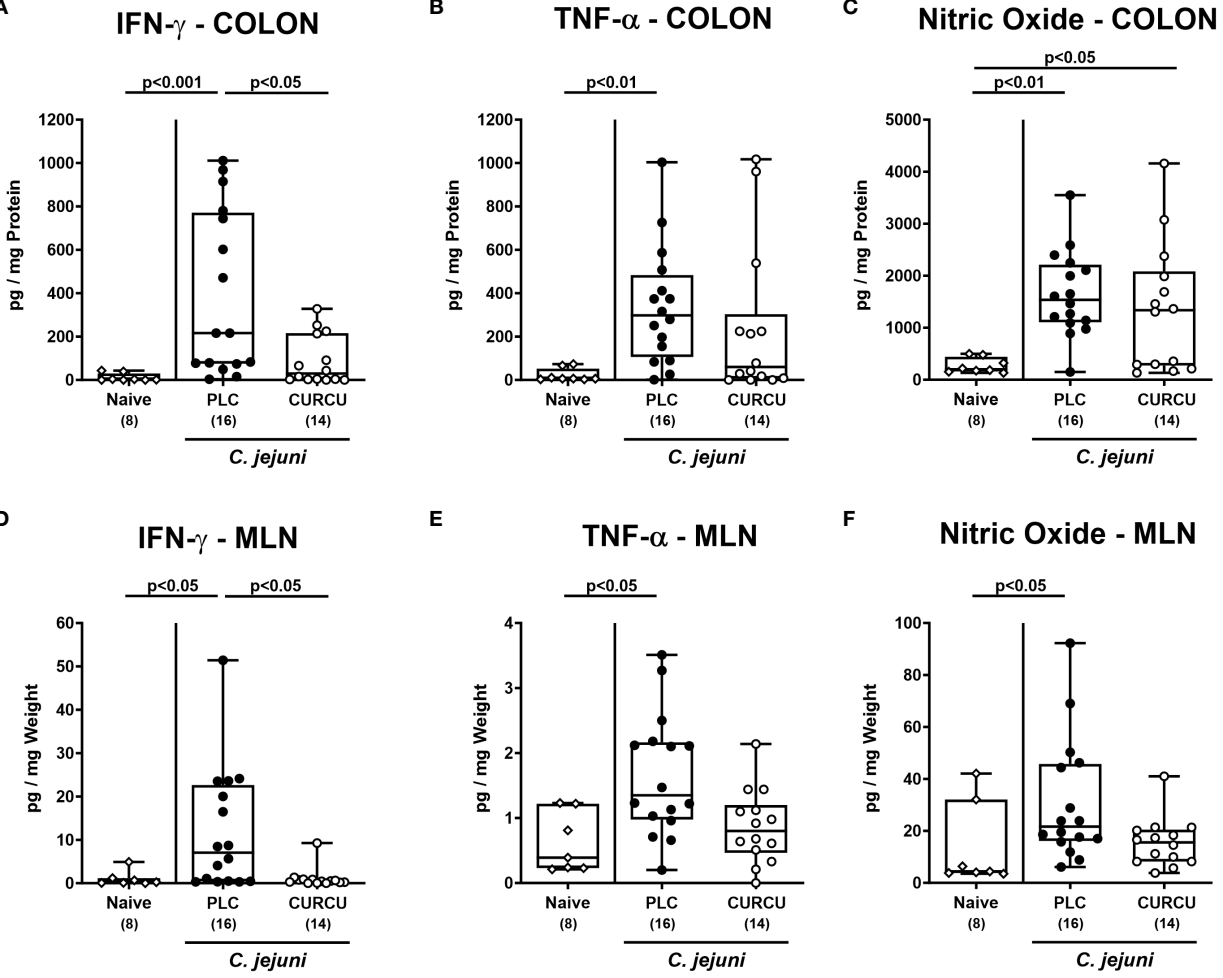
Intestinal pro-inflammatory mediators following curcumin pretreatment of *C. jejuni* infected mice. Secondary abiotic IL-10^-/-^ mice were pretreated with curcumin (CURCU; open circles) or placebo (PLC; closed circles) via the drinking water starting 7 days prior peroral infection with *C. jejuni* 81–176 strain on days 0 and 1. **(A, D)** IFN-γ, **(B, E)** TNF-α, and **(C, F)** nitric oxide concentrations were measured in supernatants of *ex vivo* biopsies derived from the colon **(A–C)** and mesenteric lymph nodes (MLN; **D-F**) on day 6 post-infection. Naive mice (open diamonds) served as non-infected controls without prophylaxis. Box plots (25^th^ and 75^th^ percentiles), whiskers (minimum and maximum values), medians (black bar in boxes), numbers of analyzed mice pooled from 3 independent experiments (in parentheses), and significance levels (p values) determined by the Kruskal-Wallis test with Dunn’s post-hoc test **(A, B, D–F)** or by the one-sided ANOVA test with Tukey’s *post-hoc* test **(C)** are indicated.

Furthermore, we tested the effect of curcumin prophylaxis on *C. jejuni*-induced secretion of pro-inflammatory mediators. Our measurements revealed increased IFN-γ (p<0.001) and TNF-α (p<0.01) concentrations in colonic explants taken from placebo as opposed to curcumin pretreated mice on day 6 p.i. (**Figure 5A, B**), whereas colonic NO levels were comparably elevated in both infected cohorts (n.s.; **Figure 5C**). In the MLN draining the infected intestines, however, respective pro-inflammatory mediators were increased in infected mice from the placebo (p<0.05 versus naive), but not the curcumin cohort (**Figures 5D–F**). Hence, curcumin pretreatment mitigated *C. jejuni*-induced pro-inflammatory mediator secretion in the intestinal tract.

### 3.5 Colonic epithelial barrier function upon curcumin pretreatment of *C. jejuni* infected mice

**Figure 6:**
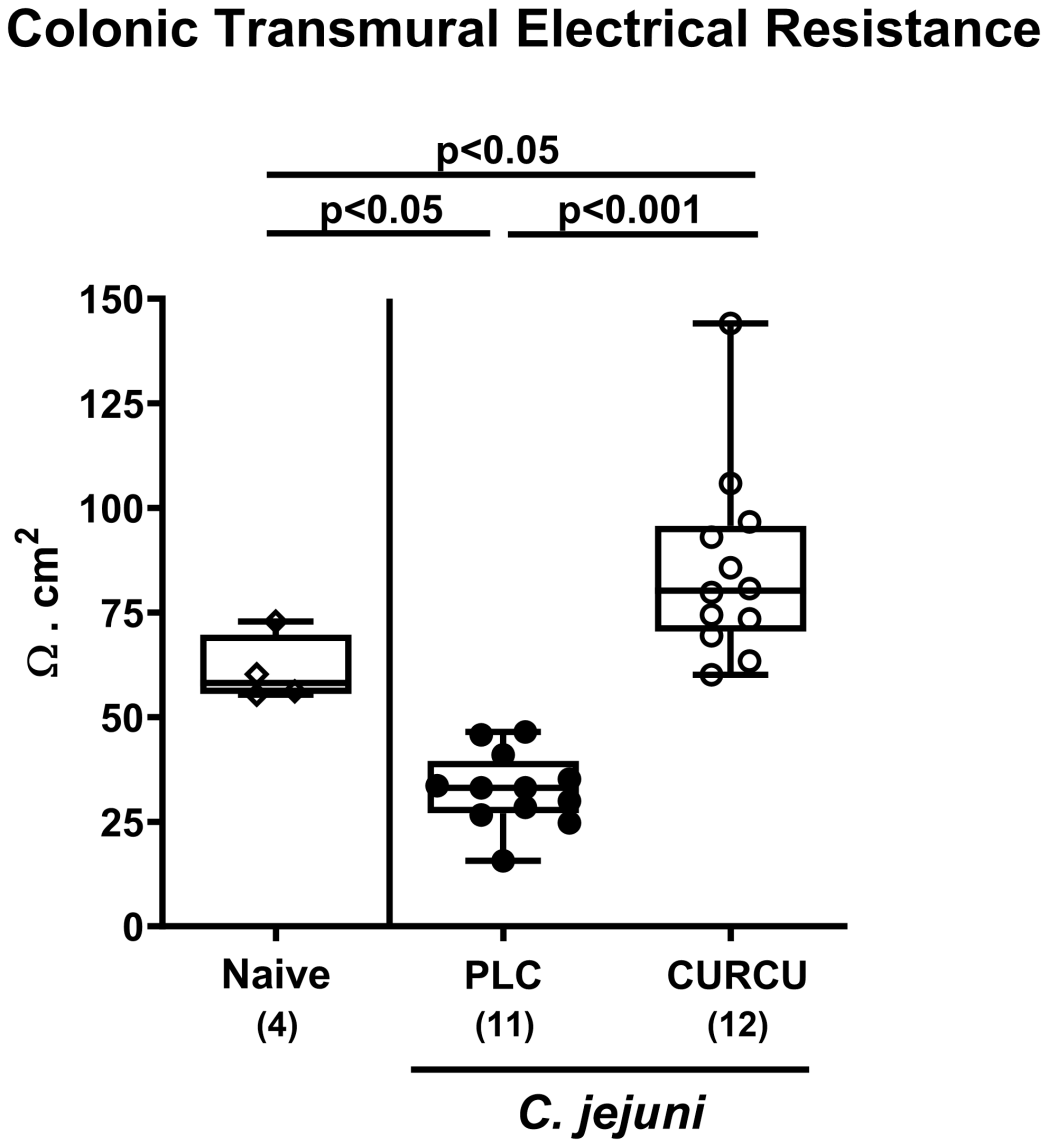
Colonic epithelial barrier function following curcumin pretreatment of *C. jejuni* infected mice. Secondary abiotic IL-10^-/-^ mice were pretreated with curcumin (CURCU; open circles) or placebo (PLC; closed circles) via the drinking water starting 7 days prior peroral infection with *C. jejuni* 81–176 strain on days 0 and 1. On day 6 post-infection, the transmural electrical resistance of the distal colon was measured in Ussing chambers. Naive mice (open diamonds) served as non-infected controls without prophylaxis. Box plots (25^th^ and 75^th^ percentiles), whiskers (minimum and maximum values), medians (black bar in boxes), numbers of analyzed mice pooled from 3 independent experiments (in parentheses), and significance levels (p values) determined by the one-sided ANOVA test with Tukey’s *post-hoc* test are indicated.

In addition, we tested whether the disease-alleviating effects upon curcumin pretreatment of *C. jejuni* infected mice had an impact on large intestinal epithelial barrier function. The electrophysiological resistance measurements of colonic *ex vivo* biopsies in the Ussing chamber on day 6 p.i. revealed that *C. jejuni* infection resulted in lower transmural electrical resistances in the colon derived from placebo, but not curcumin pretreated mice when compared to naive control animals (p<0.05; **Figure 6**). Of note, the colonic transmural resistance values were even higher in curcumin pretreated mice with induced campylobacteriosis versus non-infected and untreated controls (p<0.05; **Figure 6**). Hence, curcumin pretreatment of *C. jejuni* infected mice could rescue epithelial barrier integrity.

### 3.6 Extra-intestinal and systemic inflammatory mediator secretion following curcumin pretreatment of *C. jejuni* infected mice

**Figure 7:**
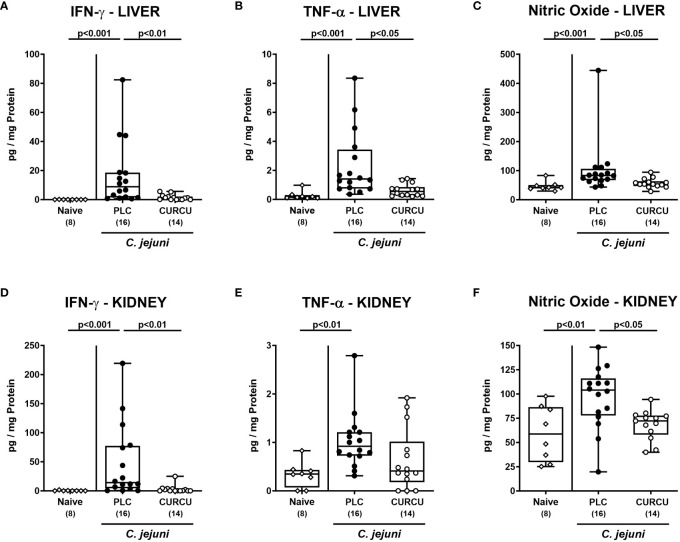
Extra-intestinal pro-inflammatory mediators following curcumin pretreatment of *C. jejuni* infected mice. Secondary abiotic IL-10^-/-^ mice were pretreated with curcumin (CURCU; open circles) or placebo (PLC; closed circles) via the drinking water starting 7 days prior peroral infection with *C. jejuni* 81–176 strain on days 0 and 1. **(A, D)** IFN-γ, **(B, E)** TNF-α, and **(C, F)** nitric oxide concentrations were measured in supernatants of *ex vivo* biopsies derived from the liver **(A–C)** and kidneys **(D–F)** on day 6 post-infection. Naive mice (open diamonds) served as non-infected controls without prophylaxis. Box plots (25^th^ and 75^th^ percentiles), whiskers (minimum and maximum values), medians (black bar in boxes), numbers of analyzed mice pooled from 3 independent experiments (in parentheses), and significance levels (p values) determined by the Kruskal-Wallis test with Dunn’s *post-hoc* test **(A–C, E, F)** or by the one sided ANOVA test with Tukey’s *post-hoc* test **(D)** are indicated.

**Figure 8:**
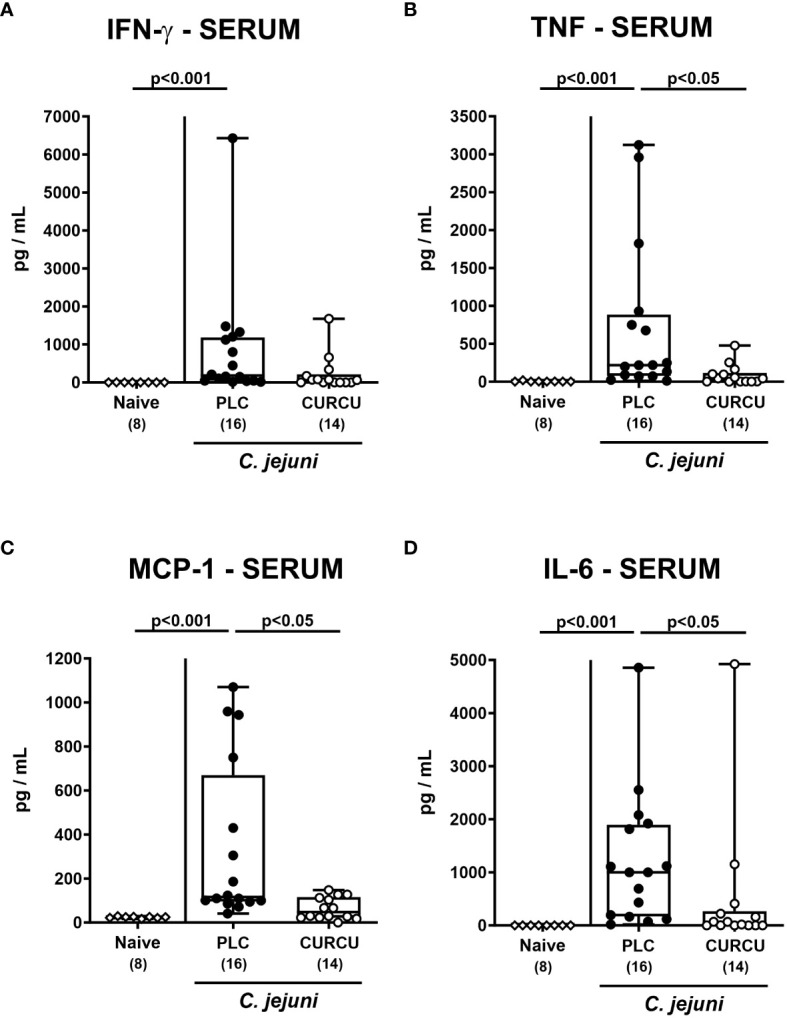
Systemic pro-inflammatory mediators following curcumin pretreatment of *C*. *jejuni* infected mice. Secondary abiotic IL-10^-/-^ mice were pretreated with curcumin (CURCU; open circles) or placebo (PLC; closed circles) via the drinking water starting 7 days prior peroral infection with *C*. *jejuni* 81–176 strain on days 0 and 1. **(A)** IFN-γ, **(B)** TNF-α, **(C)** MCP-1, and **(D)** IL-6 concentrations were measured in serum samples taken on day 6 post-infection. Naive mice (open diamonds) served as non-infected controls without prophylaxis. Box plots (25^th^ and 75^th^ percentiles), whiskers (minimum and maximum values), medians (black bar in boxes), numbers of analyzed mice pooled from 3 independent experiments (in parentheses), and significance levels (p values) determined by the Kruskal-Wallis test with Dunn’s *post-hoc* test are indicated.

We further addressed whether the anti-inflammatory properties of curcumin pretreatment were also effective in organs beyond the infected intestines. Our measurements of pro-inflammatory mediators in liver and kidney explants revealed that hepatic as well as renal IFN-γ, TNF-α, and NO concentrations were increased in placebo (p<0.01–0.001 versus naive), but not in curcumin pretreated mice on day 6 p.i. (n.s. versus naive; **Figure 7**). Strikingly, the potent inflammation-dampening effects of curcumin could also be observed systemically given that in serum samples taken 6 days following infection of curcumin-pretreated mice, basal IFN-γ, TNF-α, MCP-1, and IL-6 concentrations were measured (n.s. versus naive), whereas systemic pro-inflammatory mediators were all elevated in placebo counterparts (p<0.001 versus naive; **Figure 8**). Hence, curcumin pretreatment could mitigate also extra-intestinal and even systemic *C. jejuni*-induced pro-inflammatory mediator responses.

## 4 Discussion

In our actual preclinical placebo-controlled intervention trial, prophylactic oral curcumin application to mice starting a week before *C. jejuni* infection ameliorated the acute campylobacteriosis syndrome as evidenced by i.) an improved clinical outcome, ii.) less inflammation-induced shrinkage of the infected large intestines, iii.) less histopathological and apoptotic epithelial changes in the colon, iv.) attenuated colonic infiltration with distinct innate and adaptive immune cells, v.) diminished pro-inflammatory mediator secretion in the intestinal tract (i.e., in the colon and MLN), vi.) in extra-intestinal organs (i.e., liver and kidneys) and strikingly, vii.) even systemically if compared to placebo. In addition, viii.) the gastrointestinal *C. jejuni* numbers (i.e., in the stomach, ileum, and colon) were lower in the former versus the latter. The result of decreased gastrointestinal enteropathogen loads (**Figure 2**) were rather unexpected given that the applied curcumin concentration (1.0 g/L) was below the minimum inhibitory concentration (MIC) of 5.9 g/L (at pH 7.4). Nevertheless, one needs to take into consideration that the polyphenol is subjected to modification by distinct intestinal enzymes yielding metabolites with potential bacteria-toxic effects (21).

Apart from the lowered gastrointestinal *C. jejuni* loads the mitigated campylobacteriosis syndrome upon curcumin pretreatment (**Figure 3**) might additionally have been due to pronounced immune-modulatory effects of the polyphenol in this acute inflammatory scenario. In support, curcumin was shown to ameliorate experimental colitis of different etiologies (24, 44–52). Furthermore, our own previous work revealed that oral pretreatment of mice with the same curcumin concentration as applied in our actual trial alleviated acute *Toxoplasma gondii*-induced ileitis (23). The immune-modulatory effects of curcumin treatment were mirrored by less distinct infiltration of the infected large intestinal mucosa and lamina propria with neutrophils as well as T and B lymphocytes (**Figure 4**). In support, both *in vitro* and *in vivo* studies provided evidence that curcumin interacts with various immune cell subtypes of the innate and adaptive immune system including the afore-mentioned ones preventing from cellular infiltration of the target tissues (53). The attenuated immune cellular infiltration of the colonic tissue following curcumin pretreatment was accompanied by dampened intestinal secretion of IFN-γ, TNF-α, and NO as measured in the colon and in the MLN draining the infected intestinal tract on day 6 p.i. Remarkably, curcumin treated mice presented with colonic T and B cell numbers and furthermore, with intestinal pro-inflammatory mediator concentrations that did not differ from those detected in naive mice (**Figure 5**). Our data are well in line with previous *in vitro* and *in vivo* studies showing that curcumin was able to down-regulate the expression of respective pro-inflammatory mediators counteracting various inflammatory conditions including intestinal inflammation (53–55). In consequence of the here observed immune-modulatory effects of curcumin pretreatment, the large intestinal tissues were less distinctly exposed to cell-toxic oxidative stress resulting in less pronounced apoptotic responses in the colonic epithelial cells and in less severe histopathological damage (**Figures 3C, D**). Our data are supported by previous studies underscoring potent anti-apoptotic effects of exogenous curcumin as observed *in vitro* and *in vivo* (46, 52, 56–59). The alleviated *C. jejuni*-induced intestinal tissue damage upon curcumin pretreatment was accompanied by an uncompromised epithelial barrier function as indicated by transmural electrical resistance values measured in the colon of curcumin pretreated *C. jejuni* infected mice that were not only higher as compared to placebo counterparts, but also to naive controls (**Figure 6**), indicative for an enhanced tightening of the epithelial barrier. Our actual and previous studies further underscore potent effects of exogenous polyphenolic compounds such as curcumin and resveratrol on rescuing epithelial barrier integrity and function during acute murine campylobacteriosis (38, 60) and are supported by previous studies showing epithelial barrier-preserving capacities of curcumin application which was accompanied by up-regulated expression of tight junction proteins such as claudin-1 and zonula occludens protein-1 (ZO-1) (61–64).

Remarkably, the disease-mitigating properties of curcumin pretreatment in *C. jejuni* infected mice could not only be assessed in the intestinal tract, but also in extra-intestinal organ such as the liver and the kidneys given hepatic as well as renal IFN-γ, TNF-α, and NO concentrations in curcumin pretreated mice on day 6 p.i. that were comparable to naive values (**Figure 7**). In line with our results, curcumin was shown to exert potent anti-oxidative effects in a plethora of liver diseases as shown by enhanced radical scavenging and down-regulating the inducible nitric oxidase synthase (iNOS) which resulted in decreased hepatic NO concentrations (65, 66). Furthermore, polyphenolic application led to both, decreased hepatic TNF-α expression and attenuated cytokine-induced apoptosis (65, 66). Previous studies underlined also reno-protective properties of curcumin due to down-regulated expression of TNF-α, MCP-1, and iNOS in the kidneys as reviewed previously (67), whereas polyphenolic pretreatment attenuated renal injury in LPS-induced endotoxenemia (68). Strikingly, the disease-mitigating potency of curcumin pretreatment could also be observed systemically as shown by naive IFN-γ, TNF-α, MCP-1, and IL-6 concentrations measured in serum samples derived from *C. jejuni* infected mice of the verum cohort (**Figure 8**). In support, curcumin was shown to inhibit pro-inflammatory cytokine production in endotoxinemia (69, 70) and to prevent LPS-induced TLR-4 activation and subsequent pro-inflammatory mediator secretion by the inhibition of the TLR-4/MyD88/NF-κB signaling pathways during sepsis (71–73). The anti-TLR-4 effects of curcumin were also propagated by Lubbad and colleagues who demonstrated that curcumin treatment could ameliorate experimental colitis in a TLR-4 dependent fashion (48). Moreover, in this line, in a previous study applying an experimental *C. jejuni* infection model of an immune cell – epithelial cell co-culture, we were able to show the barrier-preserving effect of curcumin which was dependent on its inhibition of NF-κB (60).

## 5 Conclusion

Given that the hyper-activation of the host immune system by the bacterial endotoxin LOS constitutes the main molecular mechanism underlying *C. jejuni* -induced enteritis (8) and furthermore, that the risk for developing autoimmune sequelae (such as GBS, RA, and IBD) is associated with the severity of the enteritis (17), the TLR-4 antagonist curcumin constitutes an elegant antibiotic-independent strategy to mitigate acute campylobacteriosis and furthermore, to reduce the risk for post-infectious collateral damages of *C. jejuni* infection. Curcumin may be considered as non-toxic, non-mutagenic, and overall safe given that even high doses of 6 g per day for up to 7 weeks were tolerated well in human trials (74). One should also take into consideration that the systemic concentrations of the polyphenolic compound are rather low given the poor bioavailability of curcumin after ingestion (74).

## Data availability statement

The original contributions presented in the study are included in the article/supplementary material. Further inquiries can be directed to the corresponding author.

## Ethics statement

The animal study was approved by Landesamt für Gesundheit und Soziales (LaGeSo), Berlin. The study was conducted in accordance with the local legislation and institutional requirements.

## Author contributions

MH: Conceptualization, Funding acquisition, Investigation, Supervision, Validation, Visualization, Writing – original draft. SM: Investigation, Validation, Visualization, Writing – review & editing. FL: Investigation, Visualization, Writing – review & editing. EP: Investigation, Methodology, Validation, Writing – review & editing. JS: Validation, Writing – review & editing. RB: Investigation, Validation, Visualization, Writing – review & editing. SK: Investigation, Validation, Writing – review & editing. SB: Conceptualization, Funding acquisition, Supervision, Validation, Writing – review & editing.

## References

[1] WHO. World Health Organisation. Campylobacter (2020). Available online at: https://www.who.int/news-room/fact-sheets/detail/campylobacter.

[2] European Food Safety A European Centre for Disease P, Control. The european union summary report on antimicrobial resistance in zoonotic and indicator bacteria from humans, animals and food in 2019–2020. EFSA J. (2022) 20:e07209. doi: 10.2903/j.efsa.2022.7209 35382452 PMC8961508

[3] Wilson DJ Gabriel E Leatherbarrow AJ Cheesbrough J Gee S Bolton E. Tracing the source of campylobacteriosis. PloS Genet. (2008) 4:e1000203. doi: 10.1371/journal.pgen.1000203 18818764 PMC2538567

[4] Fitzgerald C. Campylobacter. Clin Lab Med. (2015) 35:289–98. doi: 10.1016/j.cll.2015.03.001 26004643

[5] Silva J Leite D Fernandes M Mena C Gibbs PA Teixeira P. *Campylobacter* spp. As a foodborne pathogen: A review. Front Microbiol. (2011) 2:200. doi: 10.3389/fmicb.2011.00200 21991264 PMC3180643

[6] Tegtmeyer N Sharafutdinov I Harrer A Esmaeili DS Linz B Backert S. *Campylobacter* virulence factors and molecular host–pathogen interactions. Fighting Campylobacter Infections: Towards One Health Approach. (2021) 431:169–202. doi: 10.1007/978-3-030-65481-8_7 33620652

[7] Cróinín T O Backert S. Host epithelial cell invasion by *campylobacter jejuni:* trigger or zipper mechanism? Front Cell Infect Microbiol. (2012) 2:25. doi: 10.3389/fcimb.2012.00025 22919617 PMC3417527

[8] Mousavi S Bereswill S Heimesaat MM. Novel clinical *campylobacter jejuni* infection models based on sensitization of mice to lipooligosaccharide, a major bacterial factor triggering innate immune responses in human campylobacteriosis. Microorganisms. (2020) 8:482. doi: 10.3390/microorganisms8040482 PMC7232424 32231139

[9] Callahan SM Dolislager CG Johnson JG. The host cellular immune response to infection by *campylobacter* spp. And its role in disease. Infect Immun. (2021) 89:e00116–21. doi: 10.1128/IAI.00116-21 PMC8281273 34031129

[10] Young KT Davis LM Dirita VJ. Campylobacter jejuni: molecular biology and pathogenesis. Nat Rev Microbiol. (2007) 5:665–79. doi: 10.1038/nrmicro1718 17703225

[11] Lobo de Sá F Schulzke J-D Bücker R. Diarrheal mechanisms and the role of intestinal barrier dysfunction in *campylobacter* infections. Curr Top Microbiol Immunol. (2021) 431:203–31. doi: 10.1007/978-3-030-65481-8_8 33620653

[12] Butkevych E Lobo de Sá FD Nattramilarasu PK Bücker R. Contribution of epithelial apoptosis and subepithelial immune responses in *campylobacter jejuni*-induced barrier disruption. Front Microbiol. (2020) 11:344. doi: 10.3389/fmicb.2020.00344 32210941 PMC7067706

[13] Kist M Bereswill S. Campylobacter jejuni. Contributions to Microbiol. (2001) 8:150–65. doi: 10.1159/000060405 11764732

[14] Backert S Tegtmeyer N Cróinín TÓ Boehm M Heimesaat MM. Chapter 1 - human campylobacteriosis. In: Klein G, editor. Campylobacter. Cambridge, MA, United States: Academic Press (2017). p. 1–25.

[15] Zautner A Johann C Strubel A Busse C Tareen A Masanta W. Seroprevalence of campylobacteriosis and relevant post-infectious sequelae. Eur J Clin Microbiol Infect Dis. (2014) 33:1019–27. doi: 10.1007/s10096-013-2040-4 PMC4013439 24413899

[16] Keithlin J Sargeant J Thomas MK Fazil A. Systematic review and meta-analysis of the proportion of *campylobacter* cases that develop chronic sequelae. BMC Public Health. (2014) 14:1203. doi: 10.1186/1471-2458-14-1203 25416162 PMC4391665

[17] Mortensen NP Kuijf ML Ang CW Schiellerup P Krogfelt KA Jacobs BC. Sialylation of *campylobacter jejuni* lipo-oligosaccharides is associated with severe gastro-enteritis and reactive arthritis. Microbes infection. (2009) 11:988–94. doi: 10.1016/j.micinf.2009.07.004 19631279

[18] Mouftah SF Cobo-Díaz JF Álvarez-Ordóñez A Elserafy M Saif NA Sadat A. High-throughput sequencing reveals genetic determinants associated with antibiotic resistance in *campylobacter* spp. From farm-to-fork. PloS One. (2021) 16:e0253797. doi: 10.1371/journal.pone.0253797 34166472 PMC8224912

[19] Goel A Kunnumakkara AB Aggarwal BB. Curcumin as "Curecumin": from kitchen to clinic. Biochem Pharmacol. (2008) 75:787–809. doi: 10.1016/j.bcp.2007.08.016 17900536

[20] Patel SS Acharya A Ray RS Agrawal R Raghuwanshi R Jain P. Cellular and molecular mechanisms of curcumin in prevention and treatment of disease. Crit Rev Food Sci Nutr. (2020) 60:887–939. doi: 10.1080/10408398.2018.1552244 30632782

[21] Scazzocchio B Minghetti L D'Archivio M. Interaction between gut microbiota and curcumin: A new key of understanding for the health effects of curcumin. Nutrients. (2020) 12:2499. doi: 10.3390/nu12092499 PMC7551052 32824993

[22] Wagle BR Donoghue AM Jesudhasan PR. Select phytochemicals reduce *campylobacter jejuni* in postharvest poultry and modulate the virulence attributes of C. Jejuni. Front Microbiol. (2021) 12:725087. doi: 10.3389/fmicb.2021.725087 34456896 PMC8397497

[23] Bereswill S Munoz M Fischer A Plickert R Haag LM Otto B. Anti-inflammatory effects of resveratrol, curcumin and simvastatin in acute small intestinal inflammation. PloS One. (2010) 5:e15099. doi: 10.1371/journal.pone.0015099 21151942 PMC2997083

[24] Sugimoto K Hanai H Tozawa K Aoshi T Uchijima M Nagata T. Curcumin prevents and ameliorates trinitrobenzene sulfonic acid–induced colitis in mice. Gastroenterology. (2002) 123:1912–22. doi: 10.1053/gast.2002.37050 12454848

[25] Sahoo DK Heilmann R Paital B Patel A Yadav VK Wong D. Oxidative stress, hormones, and effects of natural antioxidants on intestinal inflammation in inflammatory bowel disease. Front Endocrinol. (2023) 14:1217165. doi: 10.3389/fendo.2023.1217165 PMC10493311 37701897

[26] Hanai H Iida T Takeuchi K Watanabe F Maruyama Y Andoh A. Curcumin maintenance therapy for ulcerative colitis: randomized, multicenter, double-blind, placebo-controlled trial. Clin Gastroenterol Hepatol. (2006) 4:1502–6. doi: 10.1016/j.cgh.2006.08.008 17101300

[27] Singla V Pratap Mouli V Garg SK Rai T Choudhury BN Verma P. Induction with ncb-02 (Curcumin) enema for mild-to-moderate distal ulcerative colitis—a randomized, placebo-controlled, pilot study. J Crohn's Colitis. (2014) 8:208–14. doi: 10.1016/j.crohns.2013.08.006 24011514

[28] Lang A Salomon N Wu JC Kopylov U Lahat A Har-Noy O. Curcumin in combination with mesalamine induces remission in patients with mild-to-moderate ulcerative colitis in a randomized controlled trial. Clin Gastroenterol Hepatol. (2015) 13:1444–9.e1. doi: 10.1016/j.cgh.2015.02.019 25724700

[29] Bereswill S Fischer A Plickert R Haag LM Otto B Kuhl AA. Novel murine infection models provide deep insights into the "Menage a trois" of *campylobacter jejuni*, microbiota and host innate immunity. PloS One. (2011) 6:e20953. doi: 10.1371/journal.pone.0020953 21698299 PMC3115961

[30] Fiebiger U Bereswill S Heimesaat MM. Dissecting the interplay between intestinal microbiota and host immunity in health and disease: lessons learned from germfree and gnotobiotic animal models. Eur J Microbiol Immunol (Bp). (2016) 6:253–71. doi: 10.1556/1886.2016.00036 PMC5146645 27980855

[31] Warren HS Fitting C Hoff E Adib-Conquy M Beasley-Topliffe L Tesini B. Resilience to bacterial infection: difference between species could be due to proteins in serum. J Infect Dis. (2010) 201:223–32. doi: 10.1086/649557 PMC2798011 20001600

[32] Haag L-M Fischer A Otto B Plickert R Kühl AA Göbel UB. *Campylobacter jejuni* induces acute enterocolitis in gnotobiotic il-10–/– mice via toll-like-receptor-2 and-4 signaling. PloS One. (2012) 7:e40761. doi: 10.1371/journal.pone.0040761 22808254 PMC3393706

[33] Mousavi S Bereswill S Heimesaat MM. Murine models for the investigation of colonization resistance and innate immune responses in *campylobacter jejuni* infections. Curr topics Microbiol Immunol. (2021) 431:233–63. doi: 10.1007/978-3-030-65481-8_9 33620654

[34] Heimesaat MM Mousavi S Bandick R Bereswill S. *Campylobacter jejuni* infection induces acute enterocolitis in il-10-/-mice pretreated with ampicillin plus sulbactam. Eur J Microbiol Immunol. (2022) 12:73–83. doi: 10.1556/1886.2022.00014 PMC9530677 36069779

[35] Mousavi S Schmidt A-M Escher U Kittler S Kehrenberg C Thunhorst E. Carvacrol ameliorates acute campylobacteriosis in a clinical murine infection model. Gut Pathog. (2020) 12:2. doi: 10.1186/s13099-019-0343-4 31921356 PMC6947993

[36] Mousavi S Busmann LV Bandick R Shayya NW Bereswill S Heimesaat MM. Oral application of carvacrol, butyrate, ellagic acid, and 2'-fucosyl-lactose to mice suffering from acute campylobacteriosis - results from A preclinical placebo-controlled intervention study. Eur J Microbiol Immunol (Bp). (2023) 13:88–105. doi: 10.1556/1886.2023.00037 37987771 PMC10668922

[37] Heimesaat MM Mousavi S Escher U Lobo de Sá FD Peh E Schulzke J-D. Resveratrol alleviates acute *campylobacter jejuni* induced enterocolitis in a preclinical murine intervention study. Microorganisms. (2020) 8:1858. doi: 10.3390/microorganisms8121858 33255723 PMC7760181

[38] Lobo De Sá FD Heimesaat MM Bereswill S Nattramilarasu PK Schulzke J-D Bücker R. Resveratrol prevents *campylobacter jejuni-*induced leaky gut by restoring occludin and claudin-5 in the paracellular leak pathway. Front Pharmacol. (2021) 12:454. doi: 10.3389/fphar.2021.640572 PMC8082453 33935732

[39] Heimesaat MM Haag LM Fischer A Otto B Kuhl AA Gobel UB. Survey of extra-intestinal immune responses in asymptomatic long-term *campylobacter jejuni*-infected mice. Eur J Microbiol Immunol (Bp). (2013) 3:174–82. doi: 10.1556/EuJMI.3.2013.3.4 PMC3832099 24265935

[40] Heimesaat MM Alutis M Grundmann U Fischer A Tegtmeyer N Bohm M. The role of serine protease htra in acute ulcerative enterocolitis and extra-intestinal immune responses during *campylobacter jejuni* infection of gnotobiotic il-10 deficient mice. Front Cell Infect Microbiol. (2014) 4:77. doi: 10.3389/fcimb.2014.00077 24959425 PMC4050650

[41] Heimesaat MM Giladi E Kuhl AA Bereswill S Gozes I. The octapetide nap alleviates intestinal and extra-intestinal anti-inflammatory sequelae of acute experimental colitis. Peptides. (2018) 101:1–9. doi: 10.1016/j.peptides.2017.12.023 29288684

[42] Bryan NS Grisham MB. Methods to detect nitric oxide and its metabolites in biological samples. Free Radic Biol Med. (2007) 43:645–57. doi: 10.1016/j.freeradbiomed.2007.04.026 PMC2041919 17664129

[43] Heimesaat MM Bereswill S Fischer A Fuchs D Struck D Niebergall J. Gram-negative bacteria aggravate murine small intestinal th1-type immunopathology following oral infection with. Toxoplasma Gondii. J Immunol. (2006) 177:8785–95. doi: 10.4049/jimmunol.177.12.8785 17142781

[44] Zeng Z Zhan L Liao H Chen L Lv X. Curcumin improves tnbs-induced colitis in rats by inhibiting il-27 expression via the tlr4/nf-Kb signaling pathway. Planta Med. (2013) 29:102–9. doi: 10.1055/s-0032-1328057 23250811

[45] Lin Y Liu H Bu L Chen C Ye X. Review of the effects and mechanism of curcumin in the treatment of inflammatory bowel disease. Front Pharmacol. (2022) 13:908077. doi: 10.3389/fphar.2022.908077 35795556 PMC9250976

[46] Topcu-Tarladacalisir Y Akpolat M Uz YH Kizilay G Sapmaz-Metin M Cerkezkayabekir A. Effects of curcumin on apoptosis and oxidoinflammatory regulation in a rat model of acetic acid-induced colitis: the roles of C-jun N-terminal kinase and P38 mitogen-activated protein kinase. J Med Food. (2013) 16:296–305. doi: 10.1089/jmf.2012.2550 23566056

[47] Mouzaoui S Rahim I Djerdjouri B. Aminoguanidine and curcumin attenuated tumor necrosis factor (Tnf)-A-induced oxidative stress, colitis and hepatotoxicity in mice. Int Immunopharmacol. (2012) 12:302–11. doi: 10.1016/j.intimp.2011.10.010 22036766

[48] Lubbad A Oriowo MA Khan I. Curcumin attenuates inflammation through inhibition of tlr-4 receptor in experimental colitis. Mol Cell Biochem. (2009) 322:127–35. doi: 10.1007/s11010-008-9949-4 19002562

[49] Jian YT Mai GF Wang JD Zhang YL Luo RC Fang YX. Preventive and therapeutic effects of nf-kappab inhibitor curcumin in rats colitis induced by trinitrobenzene sulfonic acid. World J Gastroenterol. (2005) 11:1747–52. doi: 10.3748/wjg.v11.i12.1747 PMC4305867 15793857

[50] Deguchi Y Andoh A Inatomi O Yagi Y Bamba S Araki Y. Curcumin prevents the development of dextran sulfate sodium (Dss)-induced experimental colitis. Dig Dis Sci. (2007) 52:2993–8. doi: 10.1007/s10620-006-9138-9 17429738

[51] Arafa HM Hemeida RA El-Bahrawy AI Hamada FM. Prophylactic role of curcumin in dextran sulfate sodium (Dss)-induced ulcerative colitis murine model. Food Chem Toxicol. (2009) 47:1311–7. doi: 10.1016/j.fct.2009.03.003 19285535

[52] Zhang X Wu J Ye B Wang Q Xie X Shen H. Protective effect of curcumin on tnbs-induced intestinal inflammation is mediated through the jak/stat pathway. BMC complementary Altern Med. (2016) 16:1–11. doi: 10.1186/s12906-016-1273-z PMC4992287 27544348

[53] Haftcheshmeh SM Mirhafez SR Abedi M Heydarlou H Shakeri A Mohammadi A. Therapeutic potency of curcumin for allergic diseases: A focus on immunomodulatory actions. Biomedicine Pharmacotherapy. (2022) 154:113646. doi: 10.1016/j.biopha.2022.113646 36063645

[54] Midura-Kiela MT Radhakrishnan VM Larmonier CB Laubitz D Ghishan FK Kiela PR. Curcumin inhibits interferon-Γ Signaling in colonic epithelial cells. Am J Physiology-Gastrointestinal Liver Physiol. (2012) 302:G85–96. doi: 10.1152/ajpgi.00275.2011 PMC3345961 22038826

[55] Arshad L Haque MA Abbas Bukhari SN Jantan I. An overview of structure–activity relationship studies of curcumin analogs as antioxidant and anti-inflammatory agents. Future medicinal Chem. (2017) 9:605–26. doi: 10.4155/fmc-2016-0223 28394628

[56] Zheng L Li Y Li X Kou J Zhong Z Jiang Y. Combination of hydroxyl acetylated curcumin and ultrasound induces macrophage autophagy with anti-apoptotic and anti-lipid aggregation effects. Cell Physiol biochemistry: Int J Exp Cell physiology biochemistry Pharmacol. (2016) 39:1746–60. doi: 10.1159/000447875 27744450

[57] Park SI Lee EH Kim SR Jang YP. Anti-apoptotic effects of curcuma longa L. Extract and its curcuminoids against blue light-induced cytotoxicity in A2e-laden human retinal pigment epithelial cells. J Pharm Pharmacol. (2017) 69:334–40. doi: 10.1111/jphp.12691 28155996

[58] Banerjee S Singh SK Chowdhury I Lillard JW Jr. Singh R. Combinatorial effect of curcumin with docetaxel modulates apoptotic and cell survival molecules in prostate cancer. Front bioscience (Elite edition). (2017) 9:235. doi: 10.2741/e798 PMC5545125 28199187

[59] Loganes C Lega S Bramuzzo M Vecchi Brumatti L Piscianz E Valencic E. Curcumin anti-apoptotic action in a model of intestinal epithelial inflammatory damage. Nutrients. (2017) 9:578. doi: 10.3390/nu9060578 28587282 PMC5490557

[60] Lobo de Sa FD Butkevych E Nattramilarasu PK Fromm A Mousavi S Moos V. Curcumin mitigates immune-induced epithelial barrier dysfunction by *campylobacter jejuni* . Int J Mol Sci. (2019) 20:4830. doi: 10.3390/ijms20194830 PMC6802366 31569415

[61] Wang J Ghosh SS Ghosh S. Curcumin improves intestinal barrier function: modulation of intracellular signaling, and organization of tight junctions. Am J Physiol Cell Physiol. (2017) 312:C438–C45. doi: 10.1152/ajpcell.00235.2016 PMC5407015 28249988

[62] Hou H Qiu Y Zhao H Li D Liu Y Wang Y. Effect of curcumin on intestinal mucosal mechanical barrier in rats with non-alcoholic fatty liver disease. Zhonghua gan Zang Bing za zhi= Zhonghua Ganzangbing Zazhi= Chin J Hepatol. (2017) 25:134–8. doi: 10.3760/cma.j.issn.1007-3418.2017.02.011 PMC12814232 28297801

[63] Faralli A Shekarforoush E Ajalloueian F Mendes AC Chronakis IS. *In vitro* permeability enhancement of curcumin across caco-2 cells monolayers using electrospun xanthan-chitosan nanofibers. Carbohydr Polym. (2019) 206:38–47. doi: 10.1016/j.carbpol.2018.10.073 30553335

[64] Ghosh SS Bie J Wang J Ghosh S. Oral supplementation with non-absorbable antibiotics or curcumin attenuates western diet-induced atherosclerosis and glucose intolerance in ldlr-/- mice–role of intestinal permeability and macrophage activation. PloS One. (2014) 9:e108577. doi: 10.1371/journal.pone.0108577 25251395 PMC4177397

[65] Farzaei MH Zobeiri M Parvizi F El-Senduny FF Marmouzi I Coy-Barrera E. Curcumin in liver diseases: A systematic review of the cellular mechanisms of oxidative stress and clinical perspective. Nutrients. (2018) 10:855. doi: 10.3390/nu10070855 PMC6073929 29966389

[66] Vera-Ramirez L Pérez-Lopez P Varela-Lopez A Ramirez-Tortosa M Battino M Quiles JL. Curcumin and liver disease. Biofactors. (2013) 39:88–100. doi: 10.1002/biof.1057 23303639

[67] Trujillo J Chirino YI Molina-Jijón E Andérica-Romero AC Tapia E Pedraza-Chaverrí J. Renoprotective effect of the antioxidant curcumin: recent findings. Redox Biol. (2013) 1:448–56. doi: 10.1016/j.redox.2013.09.003 PMC3814973 24191240

[68] Chen HW Kuo HT Chai CY Ou JL Yang RC. Pretreatment of curcumin attenuates coagulopathy and renal injury in lps-induced endotoxemia. J Endotoxin Res. (2007) 13:15–23. doi: 10.1177/0968051907078605 17621542

[69] Zhang Y Liu Z Wu J Bai B Chen H Xiao Z. New md2 inhibitors derived from curcumin with improved anti-inflammatory activity. Eur J Medicinal Chem. (2018) 148:291–305. doi: 10.1016/j.ejmech.2018.02.008 29466778

[70] Wang Y Shan X Dai Y Jiang L Chen G Zhang Y. Curcumin analog L48h37 prevents lipopolysaccharide-induced tlr4 signaling pathway activation and sepsis via targeting md2. J Pharmacol Exp Ther. (2015) 353:539–50. doi: 10.1124/jpet.115.222570 25862641

[71] Zhu H-t Bian C Yuan J-c Chu W-h Xiang X Chen F. Curcumin attenuates acute inflammatory injury by inhibiting the tlr4/myd88/nf-Kb signaling pathway in experimental traumatic brain injury. J Neuroinflamm. (2014) 11:1–17. doi: 10.1186/1742-2094-11-59 PMC3986937 24669820

[72] Fu Y Gao R Cao Y Guo M Wei Z Zhou E. Curcumin attenuates inflammatory responses by suppressing tlr4-mediated nf-Kb signaling pathway in lipopolysaccharide-induced mastitis in mice. Int Immunopharmacol. (2014) 20:54–8. doi: 10.1016/j.intimp.2014.01.024 24508537

[73] Karimi A Ghodsi R Kooshki F Karimi M Asghariazar V Tarighat-Esfanjani A. Therapeutic effects of curcumin on sepsis and mechanisms of action: A systematic review of preclinical studies. Phytother Res. (2019) 33:2798–820. doi: 10.1002/ptr.6467 31429161

[74] Soleimani V Sahebkar A Hosseinzadeh H. Turmeric (Curcuma longa) and its major constituent (Curcumin) as nontoxic and safe substances: review. Phytother Res. (2018) 32:985–95. doi: 10.1002/ptr.6054 29480523

